# Longitudinal study on the seasonal variation in methylphenidate consumption in the South African private healthcare sector

**DOI:** 10.1016/j.rcsop.2025.100680

**Published:** 2025-11-03

**Authors:** Ilse Truter, Ashmitha Munasur-Naidoo

**Affiliations:** Drug Utilization Research Unit (DURU), Department of Pharmacy, PO Box 77000, Nelson Mandela University, Port Elizabeth, (Gqeberha) 6031, South Africa

**Keywords:** Attention-deficit/hyperactivity disorder (ADHD), Consumption study, Defined daily dose (DDD), Drug utilization, Methylphenidate, South Africa

## Abstract

**Background:**

A steady growth in the consumption of medicine for Attention-Deficit/Hyperactivity Disorder (ADHD) has been evident over the past two decades. The Coronavirus disease 2019 (COVID-19) pandemic is reported to have had an effect on stimulant consumption. Methylphenidate is the main active ingredient used in the treatment of ADHD, both in children and adults.

**Objective:**

The primary aim was to analyze the consumption patterns of methylphenidate in the private healthcare sector in South Africa by means of a drug utilization study using the Defined Daily Dose (DDD) methodology to establish trends, detect seasonal variations and to compare the results with international studies.

**Methods:**

A retrospective descriptive drug utilization study was conducted. IQVIA sales data from the South African private healthcare sector from 2013 to 2023 were analyzed. Consumption patterns were expressed as the number of Defined Daily Doses (DDDs)/1000 inhabitants/day and DDDs/1000 inhabitants/month. Ethical approval for the study was granted.

**Results:**

Methylphenidate consumption showed a steady increase in the years around the pandemic period, from 9.07 (in 2018) to 9.88 DDDs/1000 inhabitants/day (in 2023), with a notable lower consumption in 2020 when the COVID-19 pandemic started. Consumption in 2013 was only 6.01 DDD/1000 inhabitants/day, which indicates an increase of 64.39 % over the 11-year period. Seasonal peaks in consumption were observed in February, May, August and November, coinciding with times before assessment periods in schools and universities.

**Conclusions:**

There was a clear visible upward trend in the consumption of methylphenidate, with a decrease during 2020, as was also observed in other countries. Similar studies are recommended for other central nervous system drug classes.

## Highlights

• Methylphenidate consumption increased by 64.39 % over 11 years (2013 to 2023).
• Methylphenidate consumption decreased in 2020 during the COVID-19 pandemic.
• Seasonal peaks and troughs in consumption coincided with academic school calendars.

## 1 Introduction

Attention-Deficit/Hyperactivity Disorder (ADHD) is a common childhood neurodevelopmental disorder, characterized by impairing levels of inattention and/or hyperactivity-impulsivity.^1^ The symptoms, which impair social, academic and/or occupational functioning, start to appear before the age of 7 years and are observed in more than one setting.^2^ ADHD often lasts into adulthood.^3^ Symptoms might look different in older ages. For example, in adults hyperactivity may appear as extreme restlessness or wearing others out with their activity.^3^

Methylphenidate is the most commonly prescribed drug for ADHD in many countries.^4^ Methylphenidate is approved for the treatment of ADHD in adults and children 6 years and older.^5^^,^^6^ Methylphenidate serves as a second-line therapy for narcolepsy in adults.^6^ Other off-label uses include cancer-related fatigue, refractory depression in older adults, apathy in patients with Alzheimer's disease and cognitive enhancement (for example, memory improvement); the efficacy of methylphenidate for these conditions is reported to be moderate at best.^5^ In South Africa, methylphenidate is used mainly for the treatment of Attention-Deficit/Hyperactivity Disorder (ADHD) and narcolepsy.^6^ Methylphenidate was initially the only stimulant drug available for these purposes in South Africa,^7^ but a second stimulant (lisdexamphetamine) has since been added for ADHD. Lisdexamphetamine is indicated for ADHD only after failed therapy with methylphenidate and atomoxetine.^6^ Methylphenidate is classified as a Schedule VI substance that may have a moderate to high potential for abuse or for producing dependence.^8^ Methylphenidate use is therefore strictly controlled.

A steady growth in the consumption of medicine for ADHD has been evident over the past few decades.^8^^,^^9^ The growth can be attributed to an increased awareness of ADHD, the broadening of the diagnostic criteria for ADHD and the increased focus on ADHD in adults.^8^ A study conducted among adults in Iceland reported that consumption increased in 2003 from 2.9 DDDs/1000 inhabitants/day to 12.2 DDDs/1000 inhabitants/day in 2012.^9^ A study on data from the Norwegian Prescription Database from 2006 to 2022, found that the overall prevalence of ADHD medication use in 6- to 64-year-olds increased from 5.2 to 19.4 per 1000 in the period, most pronounced from 2020 and onwards.^10^ A study published by the UN Narcotics Control Board (1994–1996) reported 0.12 DDDs/1000 inhabitants/day for methylphenidate.^11^ In the private healthcare sector of South Africa, consumption of methylphenidate was 6.010 DDDs/1000 inhabitants/day in 2013, and 7.827 DDDs/1000 inhabitants/day in 2016.^12^

The Coronavirus disease 2019 (COVID-19) pandemic also had an influence on stimulant consumption. Silva and colleagues^13^ reported that following the start of strict anti-pandemic measures, from March 2020 to May 2021 in Portugal, a 9 % lower than expected prescription rate for medication for ADHD was observed, while from June 2021 to December 2023, the prescription rate was greater than expected (+32 %, with a peak of +41 % in children 5 to 9 years old) and more pronounced in girls.^13^ A retrospective cohort study^14^ in two international centers of different origins and cultures, one in Europe (Italy) and one in Central America (Costa Rica), was conducted to assess the impact of the COVID-19 pandemic on ADHD medication prescriptions and costs. It was found that from 2019 to 2022, both methylphenidate and atomoxetine prescriptions grew steadily, confirming a much higher incidence of the condition than in pre-pandemic periods.^14^ The study showed that the global pandemic had an influence on the increase in the number of ADHD medication prescriptions.^14^ Grimach and others^15^ conducted a study in 47 countries, and reported that in 2021, most countries recorded higher ADHD medication use than predicted at the end of 2019. On average, their study found that consumption increased per country by 1.60 %.^15^

For longitudinal studies, the World Health Organization (WHO) Anatomical Therapeutic Classification/Defined Daily Dose (ATC)/DDD) methodology^16^ is the preferred reporting system. Grimach and others^15^ also used the same methodology in their study. The primary aim of the study was therefore to analyze the consumption patterns of methylphenidate in the private healthcare sector in South Africa by means of a drug utilization study using the Defined Daily Dose (DDD) methodology to establish trends, detect seasonal variations and to compare the results with international studies.

## 2 Methods

### 2.1 Study design

A retrospective descriptive drug utilization study was conducted covering a period of 11 years (2013 to 2023). The study was a follow-up study of a previous South African study^12^ that used the same methodology over the period 2013 to 2016. Annual and monthly trends were analyzed, with specific emphasis on what happened during 2020 when the COVID-19 pandemic started.

The IQVIA (formerly known as Quintiles and IMS Health, Inc.) database, containing the private healthcare sector medication sales per month and per year for South Africa, was used to analyze the sales data for the main medication for ADHD, namely methylphenidate (ATC code N06BA04).^16^ The database was selected, since it represents the most comprehensive validated database available in South Africa for this type of study, and since the previous study^12^ also used the same database. The reason for selecting this specific database was because it is the most comprehensive validated database available in South Africa for this type of study, and because the previous study on 2013 to 2016 data^12^ also used the same private healthcare sector database.

A drug utilization consumption study was conducted. Consumption of methylphenidate was expressed as DDDs/1000 inhabitants/day and DDDs/1000 inhabitants/month, where the number of DDDs was the total amount of the active ingredient sold in a certain time period (day or month) divided by the DDD. The DDD/1000 inhabitants/day for methylphenidate was calculated using the following formula:

“Number of DDDs/1000 inhabitants/day = (number of packages dispensed x number of doses (tablets or capsules) per package x number of milligram (mg) per dose x 1000 inhabitants)

divided by

(DDD in mg x number of inhabitants in South Africa per day)”.

Similarly, the number of DDDs/1000 inhabitants/month was calculated.

The DDD for methylphenidate (ATC code N06BA04) is 30 mg oral.^16^

The data was categorized according to the European Pharmaceutical Market Research Association (EPHMRA) classification and consisted of the name of the active ingredient, trade name, dosage strength, package size and number of unit sales for each month of the six years of the study.^17^

The database only reported on private sector medication usage. The South African population was therefore based on the population statistics as published by the World Bank.^18^ In each year from 2018 to 2023 the population used is indicated in Table 1. It is generally reported that 15.7 % of the South African population belong to a private medical insurance scheme.^19^ The population in Table 1 was used as the estimated number of the population who belonged to private medical insurance schemes in the respective years of the study. For the study^12^ conducted on 2013 to 2016 data, the estimated population belonging to the private healthcare sector was 17 %.

**Table 1:** Percentage of nervous system medicine subclasses sold from 2018 to 2023.

Anatomical group⁎	Year 2018	Year 2019	Year 2020	Year 2021	Year 2022	Year 2023	AVERAGE
	**(*n*** **=** **91,009,779)**	**(*n*** **=** **95,717,381)**	**(*n*** **=** **96,772,971)**	**(*n*** **=** **106,538,858)**	**(*n*** **=** **105,352,488)**	**(n** **=** **106,494,079)**	**(*n*** **=** **100,314,259)**
N1 Anaesthetics	2.14	2.02	1.84	2.02	1.61	1.19	1.79
N2 Analgesics	68.56	68.59	68.28	69.95	69.30	69.44	69.05
N3 Anti-epileptics	3.47	3.49	3.50	3.29	3.59	3.60	3.49
N4 Anti-parkinson preparations	0.32	0.33	0.34	0.32	0.33	0.32	0.33
N5 Psycholeptics	13.28	13.29	13.82	12.92	13.29	13.21	13.29
N6 Psychoanaleptics	10.43	10.38	10.31	9.91	10.23	10.51	10.29
N7 Other Central Nervous System drugs	1.81	1.91	1.90	1.59	1.66	1.73	1.76
**N TOTAL NERVOUS SYSTEM**	**100.00**	**100.00**	**100.00**	**100.00**	**100.00**	**100.00**	**100.00**

⁎ The European Pharmaceutical Market Research Association (EPHMRA) classification was used in the table. The EPHMRA and ATC classifications are slightly different.

### 2.2 Methylphenidate products in South Africa

Prescribing in the South African public sector is according to the Essential Medicine List (EML).^20^ Short-acting methylphenidate is included in the Pediatric Hospital Level Standard Treatment Guidelines (STGs) and EML.^20^ In the public sector therefore, methylphenidate is only listed for children and adolescents (and not for adults). The authors know anecdotally that methylphenidate is used in the public sector but despite attempts, no reliable consumption figures for methylphenidate could be obtained for the public healthcare sector. The focus was therefore only on methylphenidate in the private healthcare sector, and hence only the private sector population was reported on.

Three other stimulants that can be used for ADHD were not included, namely lisdexamfetamine (ATC code N06BA12) dexamphetamine (ATC code N06BA01) and pemoline (ATC code N06BA05). Lisdexamfetamine was introduced in May 2021 in South Africa and only 118,764 products had been sold over the 2.5 years. Lisdexamfetamine, also used for binge eating disorder, should only be considered when treatment with methylphenidate and atomoxetine has failed. Dexamphetamine was introduced in March 2022 and only 14,875 products had been sold in the first 14 months. Pemoline has been discontinued in 2005 in South Africa due to concerns about hepatotoxicity.^21^ Atomoxetine, which is also used in ADHD, is a non-stimulant and was not included. Atomoxetine is not currently included in the South African EML.^20^

The study therefore reports on the consumption of methylphenidate using only the private sector population (15.7 % of the population). Microsoft Excel® was used for statistical analysis. Basic descriptive statistics were calculated.

### 2.3 Data validation

The IQVIA dataset adheres, to the best of the authors knowledge, to the European Union FAIR (Findable, Accessible, Interoperable, and Reusable) Criteria,^22^ as well as the South African Protection of Personal Information (POPI) Act.^23^ Both authors independently determined descriptive statistics for the data and then compared the results to ensure accuracy.

### 2.4 Ethics approval

Ethical approval for the study was obtained from the Nelson Mandela University Research Ethics Committee (Human) (registration number: H22-HEA-PHA-001). Only aggregate sales data was used. No patient identifiers were available in the data.

## 3 Results

### 3.1 Consumption of nervous system medicines

The consumption of the different subclasses of nervous system medicines in the private healthcare sector for the study period is given in Table 1. Analgesics were the most often prescribed subclass of nervous system medicines (average of 69.05 % of all products sold over the six years), followed by psycholeptics (13.29 %) and psychoanaleptics (10.29 %). Methylphenidate is classified under psychoanaleptics (N6).

### 3.2 Methylphenidate sales according to number of packages from 2018 to 2023

**Fig. 1:**
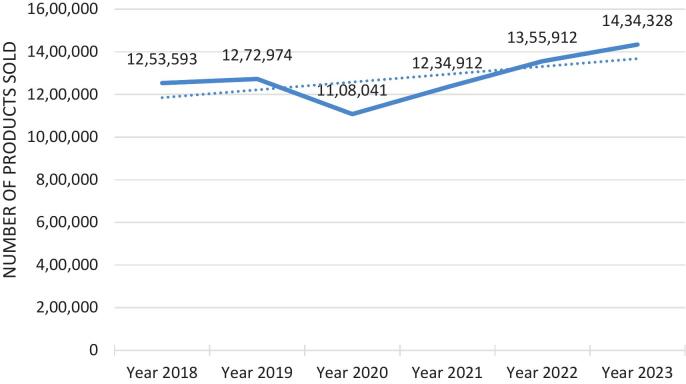
Number of methylphenidate packages over the six-year period.

The number of methylphenidate products (packages) sold over the six-year period is shown in Fig. 1. There was a general increase over the six-year period as can be seen from the trend line, however, a clear decrease can be observed in the year 2020. The COVID-19 pandemic with the associated lockdowns started in 2020.

### 3.3 Methylphenidate trade names and dosage strengths

There were 12 different trade names available for methylphenidate, in 44 different product packages (different dosage strengths), during the period from 2016 to 2023, as follows:

• Acerta XR® (18 mg, 27 mg, 36 mg, 54 mg tablets)
• Concerta® (18 mg, 27 mg, 36 mg, 54 mg tablets)
• Contramyl XR® (18 mg, 27 mg, 36 mg, 54 mg tablets)
• Medikinet MR® (5 mg, 10 mg, 20 mg, 30 mg, 40 mg capsules)
• Mefedinel OROS® (18 mg, 27 mg, 36 mg, 54 mg capsules)
• Meglarat PR® (27 mg, 36 mg, 54 mg tablets)
• Methylphenidate BIO® (10 mg tablets)
• Methylphenidate UN PR® (18 mg, 27 mg, 36 mg, 54 mg)
• Methylphenidate® (10 mg tablets)
• Neucon OROS® (18 mg, 27 mg, 36 mg, 54 mg)
• Radd XR® (18 mg, 27 mg, 36 mg, 54 mg tablets)
• Ritalin® 10 mg tablets and Ritalin® SR 20 tablets, and Ritalin LA® (10 mg, 20 mg, 30 mg, 40 mg capsules)

**Fig. 2:**
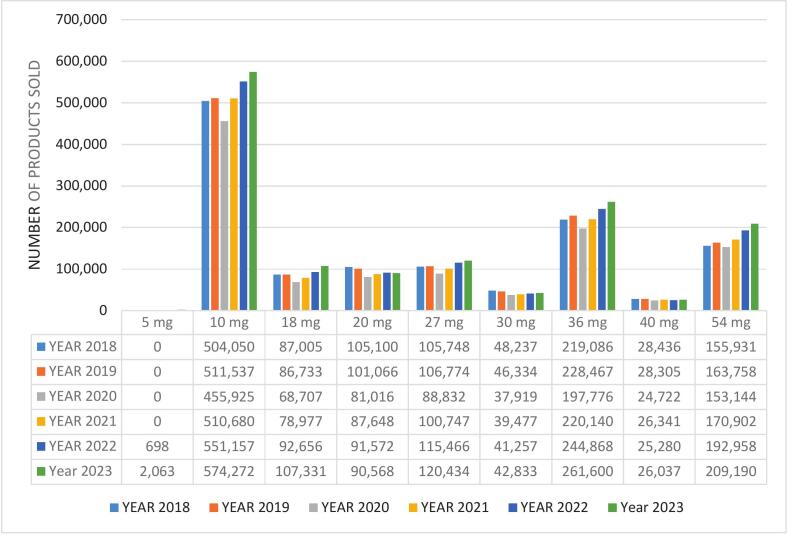
Number of methylphenidate packages according to dosage strength from 2018 to 2023.

The number of methylphenidate packages according to dosage strength over the six-year period is shown in Fig. 2.

### 3.4 Consumption of methylphenidate according to the DDD methodology

Consumption of methylphenidate, expressed as DDDs/1000 Inhabitants/day is shown in Table 2 for the period from 2018 to 2023. Both the number of products (packages sold) as well as the consumption expressed in DDDs have increased over the study period.

**Table 2:** Methylphenidate consumption expressed as DDDs/1000 inhabitants/day from 2018 to 2023.

Year	Population of South Africa	Population belonging to the private healthcare sector⁎	DDD/1000 Inhabitants/day (n⁎⁎)
2018	58,613,001	9,202,241	9.07 (*n* = 1,253,593)
2019	59,587,885	9,355,298	9.13 (n = 1,272,974)
2020	60,562,381	9,508,294	7.85 (n = 1,108,041)
2021	61,502,603	9,655,909	8.62 (n = 1,234,912)
2022	62,378,410	9,793,410	9.40 (n1 355,912)
2023	63,212,384	9,924,344	9.88 (n = 1,434,328)

⁎ 15.7 % of the South African population (the estimated population belonging to the private healthcare sector).

⁎⁎ Number of packages of methylphenidate.

**Fig. 3:**
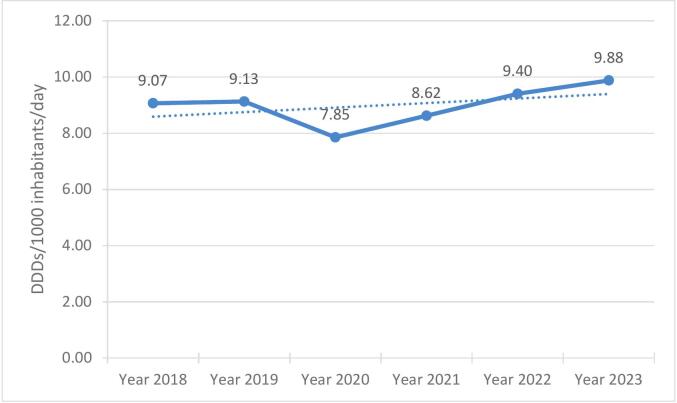
Number of DDDs/1000 inhabitants/day for methylphenidate from 2018 to 2023.

**Fig. 4:**
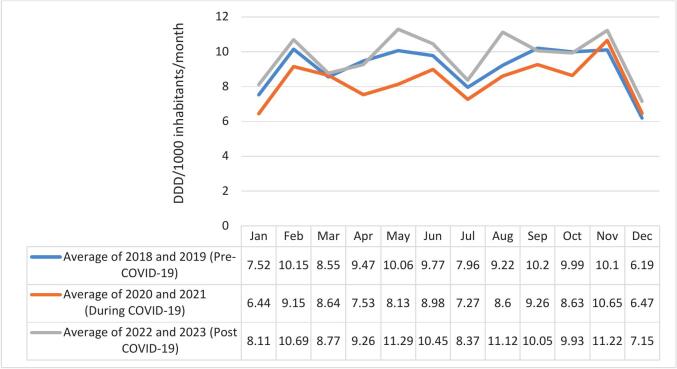
DDDs/1000 inhabitants/month for methylphenidate from 2018 to 2023, before, during and after the COVID-19 pandemic.

Fig. 3 shows the number of DDDs/1000 inhabitants/**day** for methylphenidate from 2018 to 2023. Fig. 4 shows the number of DDDs/1000 inhabitants/**month** for methylphenidate from 2018 to 2023 before (2018–2019), during (2020−2021) and after (2022−2023) the COVID-19 pandemic.

**Fig. 5:**
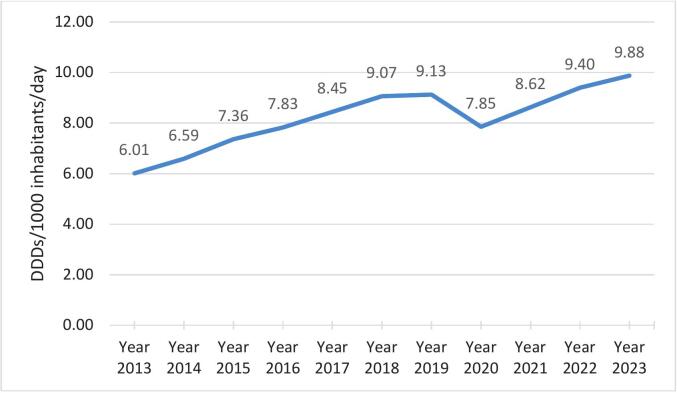
DDDs/1000 inhabitants/day for methylphenidate from 2013 to 2023*. *Mean imputation was used for the year 2017, since only data for the first 6 months of 2017 was available.

If the period before the COVID-19 pandemic is used as a “benchmark” (blue line in Fig. 5), it is clear that less methylphenidate was sold during the pandemic in the private healthcare sector (orange line in Fig. 5), but after the pandemic (grey line in Fig. 5), more methylphenidate was sold during certain months (the months that methylphenidate are expected to peak in South Africa).

The DDDs/1000 inhabitants/month for methylphenidate showed very similar patterns for Year 2018 and Year 2022 (these two years can be considered “normal years”, the years before and after the COVID-19 pandemic). Similarly, Year 2019 and Year 2021 showed different but comparable patterns (the year when news about the pandemic broke, and the year when the pandemic was in a sense brought under control). The monthly pattern in Year 2020, when the pandemic peaked, showed the greatest deviation. Year 2023 had especially higher peaks in May and August.

### 3.5 Comparison with a previous study

A similar study^12^ was conducted on methylphenidate sales figures from 2013 to 2016. Only three trade names of methylphenidate were available then. The available short- and long-acting methylphenidate-containing products in South Africa from 2013 to 2016 study^12^ were (10 products):

• Concerta® (18 mg, 27 mg, 36 mg, 54 mg tablets)
• Methylphenidate HCl-Douglas® (10 mg tablets)
• Ritalin® (10 mg tablets), Ritalin LA® (10 mg, 20 mg, 30 mg, 40 mg capsules)

The number of DDDs/1000 inhabitants/day for methylphenidate from 2013 to 2016 is given in Table 3.

**Table 3:** Methylphenidate consumption expressed as DDDs/1000 inhabitants/day from 2013 to 2016^12^.

Year	Population of South Africa	Population belonging to the private healthcare sector⁎	DDDs/1000 Inhabitants/day (n)
2013	52,980,000	9,006,600	6.01 (*n* = 827,359)
2014	54,000,000	9,180,000	6.59 (*n* = 920,920)
2015	54,960,000	9,343,200	7.36 (*n* = 1,036,293)
2016	55,910,000	9,504,700	7.83 (n = 1,126,558)

⁎ 17 % of the South African population (the estimated population belonging to the private healthcare sector).

Combining the results from the two studies, Fig. 5 shows the combined DDDs/1000 inhabitants/month for methylphenidate from the current study and the previous study.

## 4 Discussion

The first-line treatment for ADHD in South Africa is methylphenidate.^6^ There is a wide variety of methylphenidate products available on the South African market. New trade name products of methylphenidate in new and innovative dosage formulations and different dosage strengths became available during the last number of years. Only four trade name products were available in the 2013 to 2016 study^12^ (with 10 different products based on dosage formulation and strength), compared to 12 trade name products in the 2018 to 2023 study (with 44 different products based on dosage formulation and strength).

The DDD for methylphenidate is 30 mg/day as recommended by the World Health Organization (WHO) for adults (the DDD for children has not been established).^16^ On average over the six-year period from 2018 to 2023, it was interesting to note that only 3.34 % of all methylphenidate products were sold in the 30 mg dosage strength. Both products in the 30 mg dosage strength were only available in capsule form, which does not allow for dosage titration. It is also interesting that there was only one 5 mg dosage strength tablet on the market (Medikinet MR® 5 mg tablets), which was only introduced in March 2022.

There are differences, apart from strength, between the different available dosage formulations. These formulations have different release mechanisms, for example extended-release (ER), osmotic release oral system (OROS), multiple-unit pellet system (MUPS®), spheroidal oral drug absorption system (SODAS®), modified release pellets, hydrophilic matrix release system and ER film coated tablets.^25^ All these dosage formulations and dosage strengths will influence the choice of a specific trade name product.

Despite the wide variety of methylphenidate products, the 10 mg dosage formulations of methylphenidate were the most popular, followed by the 36 mg and 54 mg dosage strengths. Methylphenidate 10 mg tablets (pack size 30) were the most popular product name, possibly as a result of its lower cost, medical insurance scheme formulary specifications, or clinically the option to break the tablet if dosages must be up- or down-titrated. This finding was similar to that of another South African study conducted on a community pharmacy database in which the unit sales from January 2014 to December 2022 in a corporate pharmacy group was analyzed and it was found that the 10 mg, 36 mg and 54 mg dosage strengths were also the most often dispensed.^8^ The 36 mg and 54 mg dosage strengths were OROS formulations, which is convenient due to its once-daily dosing.

Table 2 shows that the annual consumption of methylphenidate increased over the study period, and consumption was also higher. In the previous South African study,^12^ consumption of methylphenidate was 6.010 DDDs/1000 inhabitants/day in 2013, and 7.827 DDDs/1000 inhabitants/day in 2016.^12^ This finding equates to an increase of 64.39 % in consumption over the 11-year period.

There is general agreement in the literature that the consumption of methylphenidate is increasing. Khan and Hasan^24^ examined the trends in ADHD medication prescribing and explored their association with socioeconomic factors in England. The prescriptions increased significantly from 25.17 items per 1000 population in 2019/2020 (pre-COVID-19) to 41.55 items in 2023/24 (post-COVID-19), with an average annual increase of 18 % nationally. Methylphenidate remained the most prescribed medication, while lisdexamfetamine showed the highest growth rate (55 % annually, 95 % CI 40 % to 71 %, *p* < 0.01).

Gimbach and colleagues^15^ analyzed ADHD medication use in 47 countries and regions from 2014 to 2021. They investigated changes in ADHD medication consumption in 2020 and 2021 due to the impact of the pandemic mitigation measures. They also identified countries with the highest and lowest losses in the ADHD market compared to the predicted sales in 2020 and 2021 under usual, non-pandemic conditions. They also analyzed patterns in South Africa. In 2021, the inhibiting effect of the pandemic on the ADHD medicine consumption ceased. The unweighted, Winsorized mean yielded a positive deviation of 1.60 % per country compared to expectations.^15^ Only 17 of the 46 countries and regions showed lower actual medication use than predicted, with the highest ***relative*** losses in Romania (−32 %), South Africa and the Philippines (both −25 %), and highest ***absolute*** losses in South Africa (−0.55 DDD per 1000 inhabitants per day), Belgium (−0.39 DDD per 1000 inhabitants per day) and Turkey (−0.23 DDD per 1000 inhabitants per day).^15^

It is also important to consider seasonal trends in analyses. Besides similar time trends found by Gimbach and colleagues,^15^ the same seasonal pattern was observed in many countries and regions. Due to drug holidays which are mainly during the summer holiday season, medication use often drops in Quarter 3. The months differ according to the academic years in different countries. ^15^ In South Africa, the academic year starts in January and quarter 3 is therefore the period from July to September. Gimback and colleagues^15^ noted a similar pattern in 20 countries (including, South Africa), where the drop in sales was observable in Quarter 1. Although it appears as if the peak periods in the sales of methylphenidate coincide with the academic assessment periods in South Africa, causality could not be established, since experimental data on individual patients, with accurate diagnosis, was not available. Only observational data could be obtained.

The COVID-19 pandemic had an impact on many central nervous system medicine usage patterns, including psychostimulants. In the current study, a clear dip was observed in 2020, the year when the pandemic started, and strict lockdowns were introduced. Furthermore, a comparison of the peaks and troughs from 2018 to 2023 revealed that the pattern in Year 2020 was visibly different from the other years. During the lock-down period, it is known that there was limited movement of people, and many people were avoiding visits to healthcare facilities for fear of contacting the COVID-19 virus. It included pharmacies, which could explain the decrease in the sales of methylphenidate. There was a subsequent surge in prescriptions post-2021, consistent with the findings of Gimbach and colleagues,^15^ and Khan and Hassan.^24^ It may reflect the exacerbation of mental health issues during the pandemic among children and adolescents with ADHD symptoms, driven by school closures, social distancing measures, and decreased levels of physical activity.^13^

The study had several limitations. The study was conducted on aggregate sales data, descriptive statistical approaches, and was restricted to the private healthcare sector only. Diagnoses and patient demographics (such as gender, age and rural versus urban (or metropolitan) location) were not available. Without diagnoses, off-label use of methylphenidate, diversion, adherence and discontinuation could not be reported on. Similarly, drug holidays could also only be reported on by making assumptions based on aggregate analyses. The study only focused on methylphenidate, and not on other stimulants that could potentially be used for ADHD. Methylphenidate was historically mostly used by children and adolescents, however, ADHD has also become a condition of adulthood. The dosages for children are often based on weight and also up-titrated, therefore dosages are individualized. Dosages could not be investigated, since no individual dosage instructions were available for patients. Unit sales were used as a proxy for actual use. In addition, the use of drug holidays is also common for patients on methylphenidate and this could not be investigated. Future studies could, for example, include pill counts, medicine diaries or biomarkers to confirm actual use of methylphenidate. Finally, the study only focused on the private healthcare sector in South Africa and is therefore not representative of the whole of the country and does not provide a full picture of methylphenidate prescribing and use in South Africa. Despite these limitations, consumption of methylphenidate using the DDD/1000 inhabitants/day methodology allows consumption to be measured and monitored longitudinally and also allows for international comparisons to be made.

## 5 Conclusion

Methylphenidate remains the cornerstone of ADHD treatment in South Africa. Methylphenidate consumption increased over the last two decades in the private healthcare sector, despite the lower economic growth rate in the country compared to other middle-income countries. The COVID-19 pandemic had an impact on methylphenidate consumption, with a general decrease at the start of the pandemic, followed by a steeper increase following the pandemic. It is difficult to speculate on the exact reasons for this finding during 2020/2021. It was, however, the period when the pandemic was at its peak. Descriptive statistics for sales data were calculated, with no specific further data available on aspects such as social deprivation, inequalities, medicine availability, co-occurring other mental health conditions or any other possible confounding factor.

Methylphenidate also has the potential to be abused and can also be used off-label. Misuse versus legitimate prescribing cannot be determined from a study on sales data. The authors do not believe that misuse was the main reason for the steep increase in consumption after the pandemic. The importance therefore of conducting further studies on patient level data where diagnoses and dosages are available, as well as the ages of patients, cannot be over-emphasized. Possible misuse or overuse should be monitored. Two recommendations can be made, namely that a similar study be conducted in the public healthcare sector in South Africa to determine if similar patterns are observed, and secondly, that the impact of the pandemic on other central nervous system medication, especially anxiolytics and antidepressants, are also investigated, both from a sales data perspective as well as from patient-level data.

## CRediT authorship contribution statement

**Ilse Truter:** Writing – review & editing, Writing – original draft, Project administration, Methodology, Formal analysis, Data curation, Conceptualization. **Ashmitha Munasur-Naidoo:** Writing – review & editing, Methodology, Formal analysis, Conceptualization.

## Ethical approval

Ethical approval for the study was obtained from the Nelson Mandela University Research Ethics Committee (Human) (registration number: H22-HEA-PHA-001).

## Funding

This study did not receive any specific grants from funding agencies in the public, commercial or not-for-profit sectors.

## Declaration of competing interest

We hereby declare that none of the authors have anything to declare. This study did not receive any specific grants from funding agencies in the public, commercial or not-for-profit sectors.

## Data Availability

Data sharing is not applicable for this article since no new data were created or analyzed in this study. Access to the secondary dataset was provided by IQVIA.
